# Enterohaemorrhagic *Escherichia coli* activates nitrate respiration to benefit from the inflammatory response for initiation of microcolony-formation

**DOI:** 10.1186/s12866-020-01946-w

**Published:** 2020-08-20

**Authors:** Risa Nada, Shinya Ebihara, Hilo Yen, Toru Tobe

**Affiliations:** Department of Clinical Laboratory and Biomedical Sciences, Osaka University Graduate School of Medicine, 1-7 Yamadaoka, Suita, Osaka, 565-0871 Japan

**Keywords:** T3SS, Nitric oxide, Adherence, Colony formation, Anaerobic conditions

## Abstract

**Background:**

For successful colonization, enterohaemorrhagic *Escherichia coli* (EHEC) injects virulence factors, called effectors, into target cells through the type three secretion system (T3SS), which is composed of a needle and basal body. Under anaerobic conditions, the T3SS machinery remains immature and does not have a needle structure. However, activation of nitrate respiration enhances the completion of the T3SS machinery. Because nitric oxide released by the host inflammatory response increases nitrate concentration, we sought to determine the effect of the inflammatory response on initiation of EHEC microcolony-formation.

**Results:**

The colony-forming capacity was increased in accordance with the increase of nitrate in the medium. The addition of the nitric oxide-producing agent NOR-4 also enhanced the adherence capacity, which was dependent on nitrate reductase encoded by the *narGHJI* genes. Culture supernatant of epithelial cells, which was stimulated by a cytokine mixture, enhanced the colony-forming capacity of wild-type EHEC but not of the *narGHJI* mutant. Finally, colony formation by wild-type EHEC on epithelial cells, which were preincubated with heat-killed bacteria, was higher than the *narGHJI* mutant, and this effect was abolished by aminoguanidine hydrochloride, which is an iNOS (inducible nitric oxide synthase) inhibitor.

**Conclusions:**

These results indicate that the inflammatory response enhances EHEC adherence by increasing nitrate concentration.

## Background

Enterohaemorrhagic *Escherichia coli* (EHEC) is a causative agent of bloody diarrhea and hemolytic uremic syndrome. Infection of EHEC requires colony-formation onto intestinal epithelia at the large intestine after traveling through the gastrointestinal tract. The initial important step of infection is mediated by the injection of virulence factors, called effectors, through a type 3 secretion system (T3SS) [1, 2]. Effectors modulate or disrupt host cell functions and contribute to successful infection [3, 4]. One of the effectors, Tir, is localized onto the plasma membrane and becomes a receptor for an EHEC adhesin, intimin [5]. Once bacteria are attached via the intimin-Tir interaction, EHECs begin to form microcolonies on the surface of host cells and rearrange the cytoskeleton inside host cells, resulting in characteristic pathophysiological changes called A/E (attaching and effacing) lesions [6, 7]. Components of the T3SS machinery, some effectors and intimin are encoded in the chromosomal pathogenicity island LEE (locus for enterocyte effacement).

The basal body of the T3SS machinery is a macromolecular structure basically resembling the flagella basal body. The basal body consists of two rings that provide supporting structures attached to the inner and outer membranes, and the tube-like apparatus is passed through the central rings. On the basal body, the needle-like structure protrudes from the surface of the membrane [8]. The needle of the T3SS machinery of EHEC and enteropathogenic *E. coli* (EPEC) is covered by a sheath-like structure composed of EspA. EspB and EspD proteins are attached to the tip of the needle and are thought to make a pore on the host cell plasma membrane. Under anaerobic conditions, the T3SS machinery of EHEC stays in an immature complex, which lacks the needle structure. Even under anaerobic conditions, activation of specific anaerobic respiration, such as nitrate and TMAO respirations, triggers maturation by producing a needle on the T3SS machinery without increase of expression of T3S components [9].

Large intestine, which is the main target tissue of EHEC infection, is under a low concentration of oxygen at so-called physiologic hypoxia [10]. In agreement with this, the vast majority of microbes in the large intestine are obligate anaerobic bacteria belonging to the phyla Bacteroidetes and Firmicutes [11, 12]. Inflammatory reactions affect the microbial community structure by increasing facultative anaerobic bacteria and decreasing the representation of obligate anaerobic bacteria [13, 14]. Components of inflammatory response productions include reactive oxygen species (ROS) and reactive nitrogen species (NRS), which affect changes in microbial community structure. Nitric oxide is produced by inducible nitric oxide synthase (iNOS) and converted to nitrate after reaction with superoxide radicals (O_2_^-^) [15]. Nitrate levels in mouse intestine at non-stimulated conditions are lower than detectable levels but DSS-induced colitis greatly increases to the level over 0.3 mM, which is dampened by aminoguanidine treatment [16]. The increase in nitrate by the inflammatory response has been shown to stimulate the growth of the commensal bacterium *E. coli*, which is dependent on nitrate respiratory activity [16]. Because infection of epithelial cells with pathogens induces an inflammatory response [17], the initial infection of even a small number of pathogens could accelerate the growth of pathogens through an increase in nitrate concentration.

Under anaerobic conditions, the majority of EHECs produce T3SS machinery without a needle structure [9], which is not ready for infection. We hypothesized that even low levels of EHEC initiating infection inflammatory responses would change the microenvironment by producing nitric oxide, which increases the EHEC possessing mature T3SS machinery and accelerates colony formation. We explored the effect of nitric oxide and factors secreted by epithelial cells in response to inflammation on the capacity of EHEC adherence to initiate microcolony-formation.

## Results

### Enhancement of T3SS activity by nitrate

**Fig. 1:**
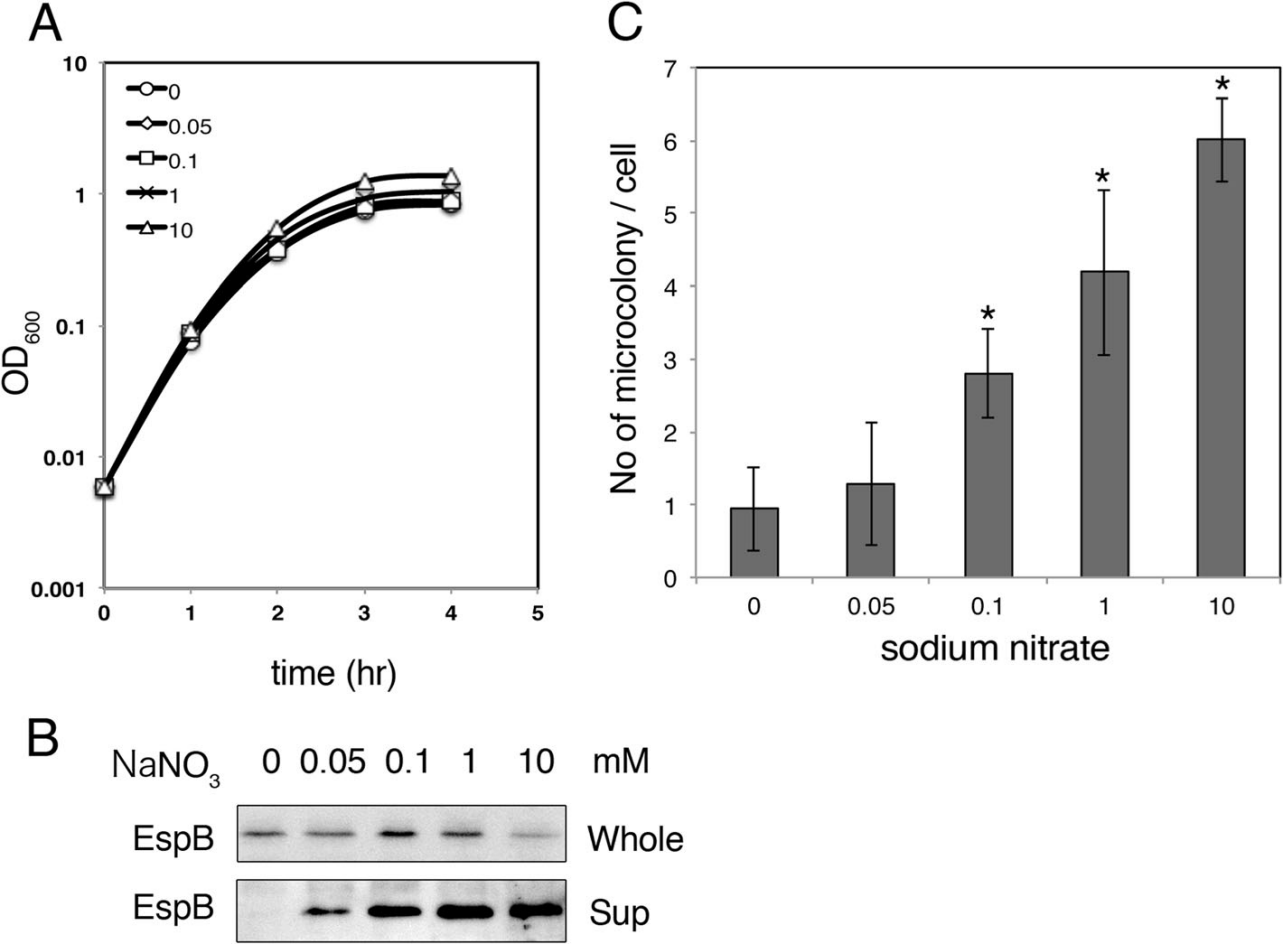
Enhancement of T3S and colony formation by nitrate. EHEC O157 Sakai wild-type strains were grown in LB containing various concentrations of sodium nitrate under microaerobic conditions at 37 °C. **a**. Growth of EHEC in the media containing various concentration of sodium nitrate (mM). Cell density was measured by OD_600_ every 1 h. The representative of three independent experiments is shown. **b**. Secretion of EspB by EHEC. Proteins in culture supernatant (Sup) were collected from 5 h post-inoculation by TCA precipitation and dissolved in SDS-sample buffer as normalized by OD_600_ of culture. Whole cell lysate (Whole) was prepared from 0.5 ml culture by collecting bacteria and dissolved in SDS-sample buffer as normalized by OD_600_. EspB protein was detected by immunoblotting with anti-EspB antibody. **c**. Microcolony formation on HeLa cells. Bacterial culture at 3 h post-inoculation was used for the infection of HeLa cells. Microcolonies formed at 3.5 h after washing at 1.5 h post-infection were determined by staining cells with Giemsa. A colony with at least 8 bacteria was counted as a microcolony. The average of three independent experiments along with standard error is shown. *: *p* < 0.05

In our previous study, maturation of the T3SS machinery under anaerobic conditions was achieved by the activation of specific anaerobic respiration using an electron acceptor as nitrate. To show the dependency of T3SS function and adherence capacity on nitrate, an EHEC wild-type strain was grown in LB medium containing various concentrations of nitrate under microanaerobic conditions at 37 °C. As expected, the growth of EHEC was enhanced and reached a higher density in accord with the increase in the concentration of nitrate (Fig. 1a). To examine the maturation of T3SS, we compared the secretion of the effector protein EspB into the culture supernatant (Fig. 1b). Although EspB protein was produced in EHEC grown without nitrate (see Fig. 1b, 0 mM), EspB in culture supernatant was barely detected. The EspB protein level in the supernatant was increased in accordance with the increase in nitrate (Fig. 1b). Because EspB protein in whole cells was not increased by an increase in nitrate, this clearly indicates that T3SS function is activated by the presence of nitrate and that activity is dependent on the nitrate concentration. T3SS function is necessary for intimate attachment and hence microcolony formation on epithelial cells [18]. The effect of various concentrations of nitrate on the efficiency of microcolony formation, which reflects the adherence capacity of cultured bacteria, was examined. After growth in LB medium containing various concentrations of nitrate, HeLa cells were infected for 90 min with the same number of bacteria (1 × 10^7^ cfu) and further cultivated after washing out uninfected bacteria for 3.5 h. During this period, EHEC formed microcolonies originated from intimately attached single bacteria, whose adherence is dependent on T3SS function. Finally, the cells were washed and stained, and the number of microcolonies on HeLa cells was compared. The number of microcolonies per cell, which corresponds to number of intimately attached bacteria at the initial infection period, was increased in accordance with the increase in nitrate in preculture medium (Fig. 1c). Importantly, even at a low concentration of nitrate (100 μM), the effect was clearly shown. These results indicated that an increase in nitrate stimulates the activity of T3SS and the capacity for colony formation, as shown previously.

### Nitric oxide increases the capacity of colony formation

**Fig. 2:**
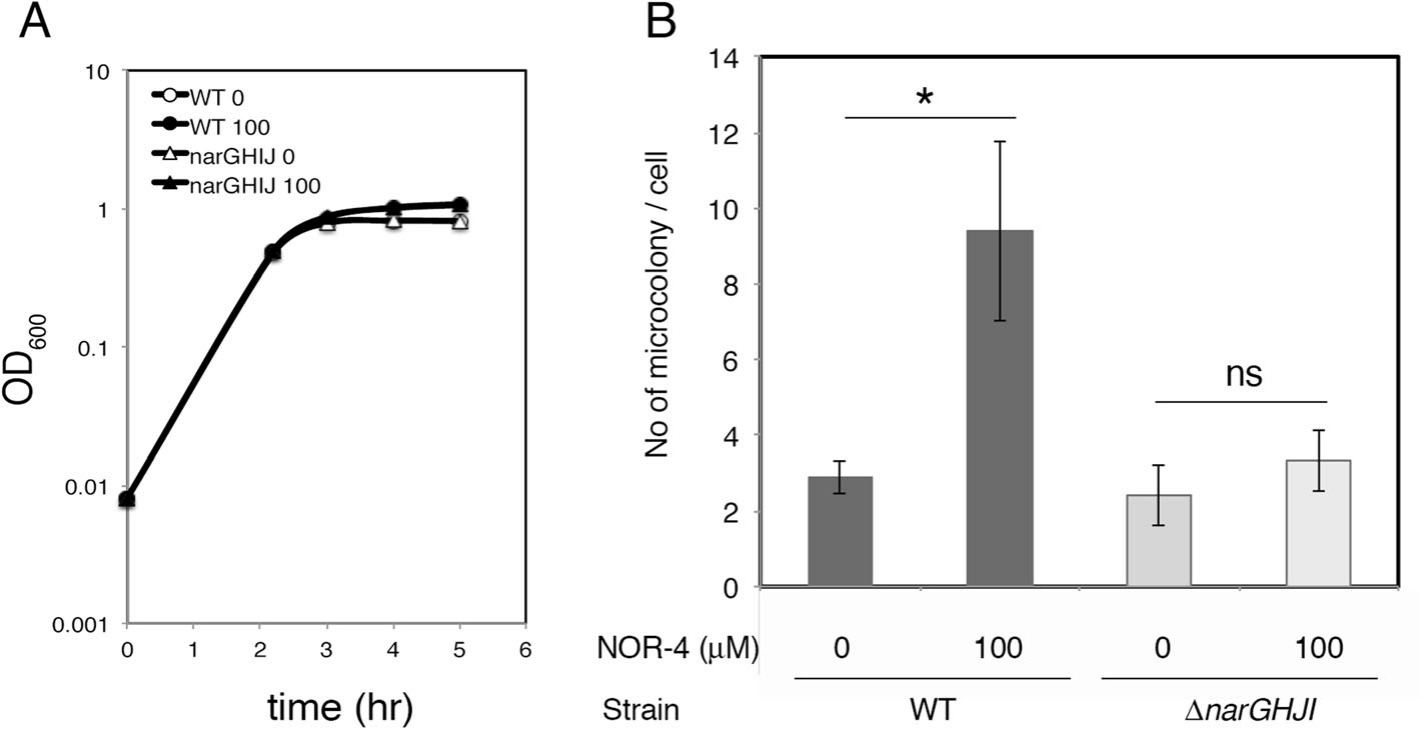
Effect of nitric oxide production on EHEC’s colony-forming capacity. EHEC O157 Sakai wild-type (WT) or the *narGHJI* deletion mutant (Δ*narGHJI*) were grown for 3 h at 37 °C in LB pretreated with (100 μM) or without (0) NOR-4 and used to infect HeLa cells for 5 h, including 3.5 h post-washing. **a**. Growth of EHEC strains in the presence/absence of NOR-4. The representative of three independent experiments is shown. **b**. Effect of NOR-4-containing medium on the colony-forming capacity. Microcolonies were visualized by staining with Giemsa. The average of three independent experiments along with the standard error is shown. *: *p* < 0.05, ns: not significant

Nitric oxide is a source of nitrate but is also harmful to bacteria. To examine the effect of nitric oxide on T3SS activity during growth under microaerobic conditions, the nitric oxide-producing agent NOR-4 was added to the medium for bacterial culture, and the bacteria were examined for colony-forming capacity. Then, 100 μM NOR-4 was added to the medium 18 h before inoculation, and EHEC was grown for 3 h under microaerobic conditions. The growth of EHEC in the presence of NOR-4 was not repressed but rather stimulated (Fig. 2a). The colony-forming frequency of EHEC was increased 3-fold compared to EHEC grown without NOR-4. Because maturation of the T3SS machinery by nitrate respiration is only dependent on nitrate reductase encoded by the *narGHJI* operon [9], to examine the necessity of nitrate respiration using NarGHJI nitrate reductase in enhanced colony forming capacity, the *narGHJI* mutant of EHEC was grown in medium containing NOR-4 and used for infection experiments as described previously. Although growth was stimulated by the presence of NOR-4, the colony-forming capacity of the mutant was not increased, and the capacity was comparable to the levels of wild-type and mutant EHEC grown without NOR-4 (Fig. 2b). In the presence of 100 μM NOR-4, nitrate was produced at approximately 100 μM at 18 h post addition. These results indicated that the presence of nitric oxide stimulates the EHEC colony-forming capacity through activation of T3SS machinery maturation.

### The inflammatory response alters the environment to enhance the colony-formation capacity

**Fig. 3:**
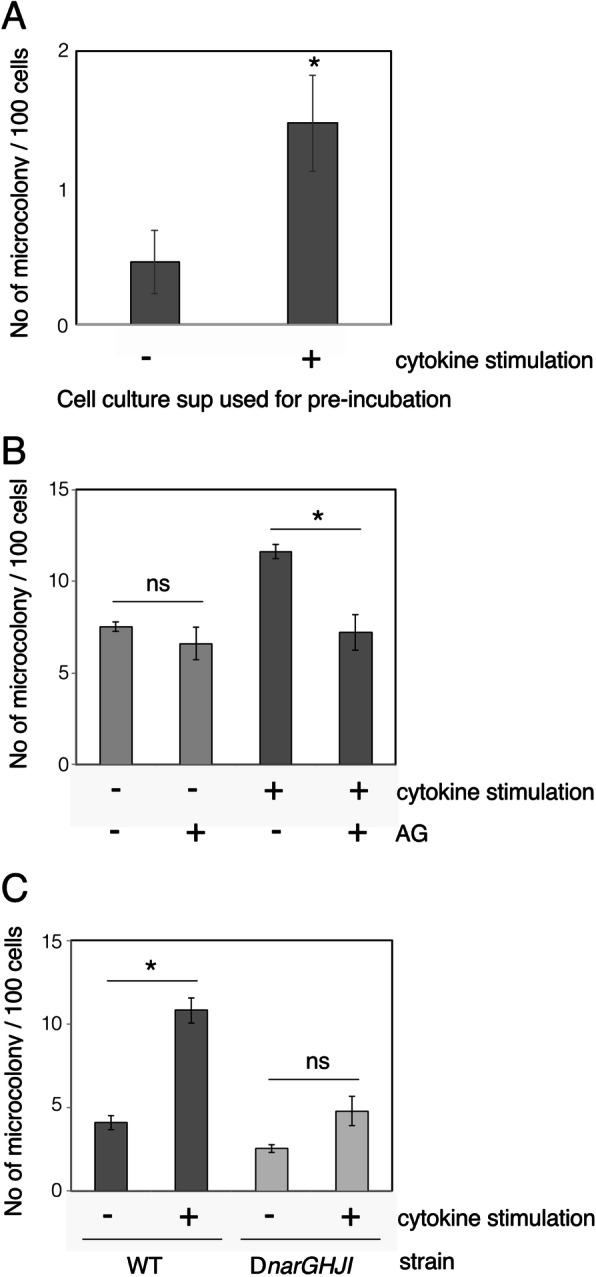
Inflammatory response-induced nitric oxide enhances EHEC’s colony-forming capacity. Caco-2 cells were exposed to a cytokine mix (IL-8, TNF-γ, IL-22) for 24 h. Culture supernatants were collected by centrifugation and filtration to remove cells and debris. EHEC O157 Sakai wild-type (WT) or the *narGHJI* mutant (Δ*narGHJI*) were grown in the supernatant for 3 h under anaerobic conditions. HeLa cells were infected with EHEC for 5 h, including 3.5 h post-washing. Microcolonies were visualized by staining with Giemsa. **a**. Effect of culture supernatant of cells in inflammation. The wild-type EHEC strain was infected into HeLa cells after growth in the supernatant. **b**. Requirement of iNOS activity for enhancement of the microcolony-forming capacity. Caco-2 cells were exposed to a cytokine mix with or without aminoguanidine hydrochloride (AG), and culture supernatants were used for pre-culture of EHEC. Microcolonies on HeLa cells were determined as in A. **c**. Activation of nitrate respiration was necessary for the enhancement. EHEC O157 Sakai wild-type (WT) or the *narGHJI* mutant (Δ*narGHJI*) were precultured in the supernatants and infected HeLa cells. The average of three independent experiments along with standard error is shown. *: *p* < 0.05, ns: not significant

The inflammatory response induces a variety of secretion factors, including nitric oxide. To explore the effect of the inflammatory response of host cells on the EHEC virulence capacity, the supernatant of cells stimulated to induce inflammation was inoculated with EHEC, and non-stimulated cells were infected. The Caco-2 cells were stimulated by a cytokine mixture (2000 U/ml IFN-γ, 50 ng/ml IL-8β, 100 ng/ml IL-22) for 24 h, and culture supernatants were collected. EHECs grown under microaerobic conditions were incubated in the culture supernatant of Caco-2 cells for 1 h, and then, HeLa cells were infected. To allow the formation of microcolonies, after 90 min of initial infection, further growth was performed after washing out unattached bacteria. The colony-forming frequency was increased when EHEC was incubated in the supernatant of cells stimulated by cytokines compared to incubation in the supernatant of non-stimulated cells (Fig. 3a). The supernatant of cytokine-stimulated cells contains a variety of factors secreted by cells. To determine the contribution of nitric oxide to the enhancement of the colony-forming capacity of EHEC, an inhibitor of iNOS activity, aminoguanidine hydrochloride (AG), was added to the Caco-2 cells along with the cytokine mix. Although the colony-forming capacity of EHEC was increased by pre-growth in the cell culture supernatant of cytokine-stimulated cells, the addition of AG abolished the effect completely (Fig. 3b). To further examine the contribution of nitric oxide to the increase of EHEC colony-forming capacity, the same infection experiments were performed with the *narGHJI* mutant of EHEC. The frequency of colony formation by the mutant was not increased even after incubation in the supernatant of cells stimulated by cytokines (Fig. 3c). The nitrate concentration of the supernatant of cells stimulated by cytokines was approximately 50–100 μM (Figure S1). These results indicated that cells involved in the inflammatory response secrete factor(s) enhancing the EHEC colony-forming capacity and that the factor was nitrate derived from nitric oxide.

### The inflammatory response enhances the adherence capacity of EHEC

**Fig. 4:**
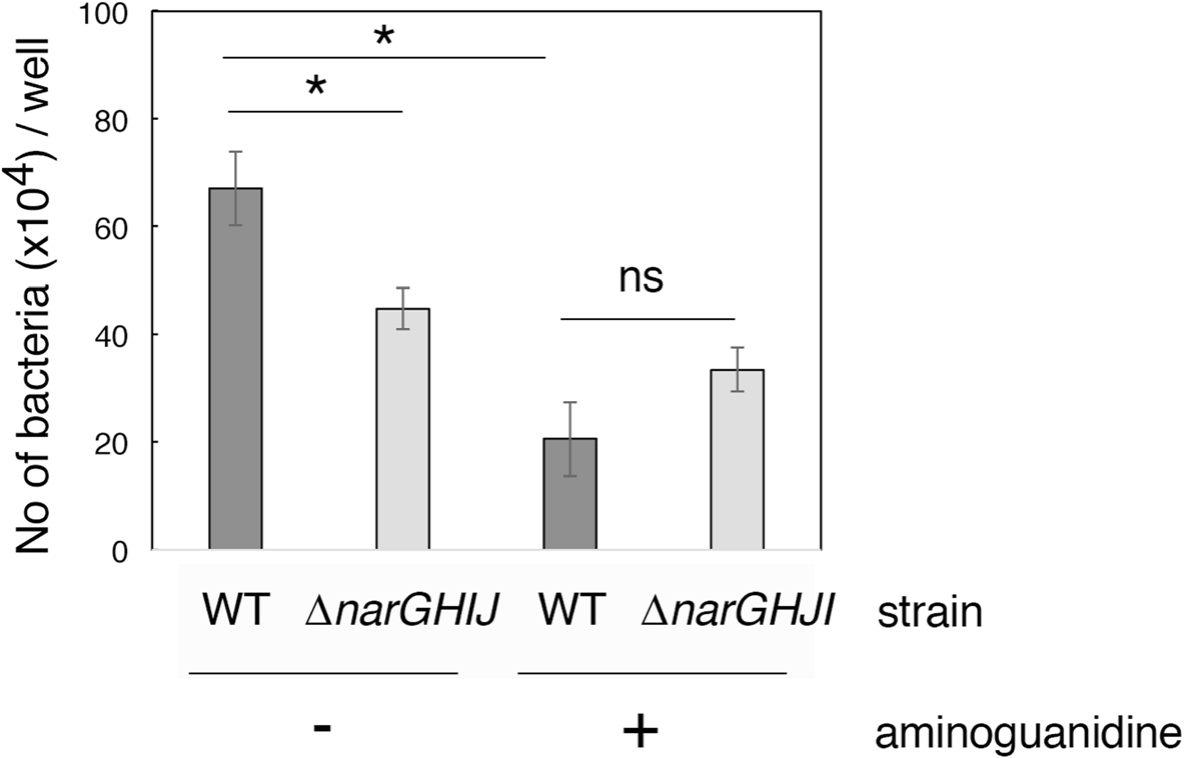
Effect of prestimulation of epithelial cells on EHEC adherence. HT-29 cells were exposed to heat-killed bacteria in the absence (−) or presence (+) of aminoguanidine hydrochloride 2 h prior to infection. EHEC O157 Sakai wild-type (WT) or the *narGHJI* mutant (Δ*narGHJI*) grown under anaerobic conditions was added to the cells to initiate infection. At 3 h post-infection, cells were washed, and adherent bacteria were recovered by lysing host cells. Viable bacterial number was determined by adequate dilution and plating. The average of three independent infections along with standard error is shown. *: *p* < 0.05, ns: not significant

The inflammatory response in host cells is induced by bacterial components when bacteria closely contact cells. This finding suggested that the microenvironment surrounding the cells changes into a nitrate-rich one that enhances EHEC T3SS activity and accelerates microcolony-formation once host cells are stimulated by bacterial attachment. To explore this hypothesis, the effect of pre-exposure of epithelial cells with bacteria on secondary infection was examined. The HT-29 cells were exposed to heat-killed EHEC for 2 h, and then, the cells were infected with EHEC wild-type or *narGHJI* mutant for 3 h. The number of adherent wild-type bacteria was greater than that of the *narGHJI* mutant (Fig. 4). The reduction of adherent bacteria was not due to the difference of growth rate, because growth of EHEC in the supernatant of the stimulated cells was not affected by the *narGHJI* mutation. This could be explained by the availability of other nitrate reductase, such as NarZYWV. To confirm the involvement of nitric oxide production, the cells were incubated with aminoguanidine hydrochloride (AG), an inhibitor of iNOS activity, while exposed to heat-killed EHEC. The number of adherent wild-type bacteria was markedly decreased to a level comparable to the *narGHJI* mutant (Fig. 4). These results indicated that cells involved in inflammation provide a microenvironment enhancing EHEC adherence by increasing the nitrate concentration.

## Discussion

**Fig. 5:**
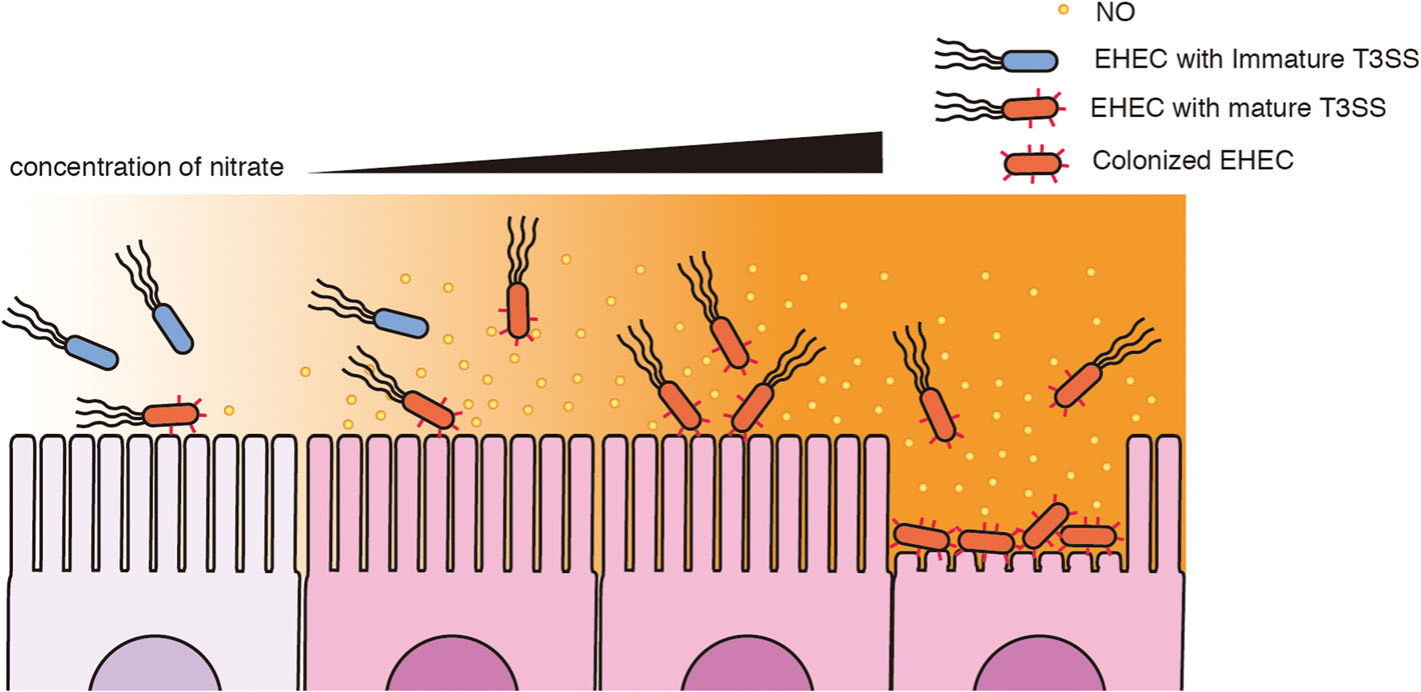
Schematic representation of nitrate-enhancing microcolony-formation of EHEC. Under hypoxic conditions in the intestine, most EHECs possess an immature form of T3SS (T3S machinery). Once epithelial cells are stimulated by interacting with T3SS-positive EHECs, cells produce nitric oxide as a part of the inflammatory response. An increase in the concentration of nitrate derived from nitric oxide enhances the maturation of T3SS in EHEC. Consequently, the efficiency of colony formation on epithelial cells is accelerated

The T3SS of EHEC plays an important role in colony-formation on epithelial cells of the intestine, which is necessary for successful infection. In hypoxic conditions, most T3SS machinery remains in an immature form and does not have a needle structure. The activation of specific anaerobic respiration has been shown to enhance the maturation of T3S machinery and its secretion activity, resulting in an enhanced adherence capacity of EHEC [9]. Among the electron acceptors EHEC can use for anaerobic respiration, nitrate can enhance T3S maturation, and only a nitrate reductase encoded by the *narGHJI* operon is involved in maturation. Because microbial infection induces an inflammatory response in epithelial cells and other host cells, the nitrate concentration around the infected area increases through the secretion of nitric oxide by host cells. We sought to examine the effect of nitric oxide produced by inflammation on T3SS activity and the adherence capacity of EHEC. The results suggested the contribution of host inflammatory response to the enhancement of adherence capacity of EHEC. This is caused by increase of nitrate concentration in the microenvironment by host cell-secreted nitric oxide (Fig. 5).

An increase in nitric oxide in the medium by the addition of NOR-4 resulted in an increase in the adherence capacity of EHEC. Since nitric oxide produced by NOR-4 quickly changed into nitrate/nitrite in water within 5 s [19], a sufficient concentration of nitrate for stimulating the growth of EHEC was provided. This finding is consistent with a previous report of the effect of NOR-4 on EHEC growth [20]. As a result, the colony-forming capacity of EHEC was enhanced by NOR-4, and this enhancement was dependent on nitrate reductase encoded by the *narGHJI* operon. The presence of NOR-4 at 100 μM indeed produced 100 μM nitrate 18 h after addition. These results clearly show that the increase of nitric oxide results in the enhancement of colony formation of EHEC through activation of nitrate respiration. The activation of iNOS by the inflammatory response has been shown to increase the nitrate concentration in the intestine and enhance the growth of *E. coli* through anaerobic nitrate respiration [16, 21]. Induction of the inflammatory response in epithelial cells with a cytokine mixture altered the culture medium to enhance the colony-forming capacity of EHEC in a nitrate-respiration-dependent manner. Furthermore, inhibition of iNOS activation while cytokine stimulation by aminoguanidine abolished the enhancing effect. These results clearly indicated that an increase in nitric oxide activates nitrate respiration and the infection capacity of EHEC.

Finally, we showed the effect of inflammatory induction on the efficiency of EHEC infection. Colony formation of the EHEC wild-type strain on epithelial cells, which were pretreated with bacterial materials, heat-killed bacteria, was higher than the *narGHJI* mutant, and this difference was diminished by the addition of the iNOS inhibitor AG. By using diffusion chamber systems, Schuller and Pillips showed that EHEC adherence in microaerobic conditions to apical surface of epithelial cells was not further increased by addition of nitrate and TMAO [22]. This could be explained by the changes of nitrate concentration at microenvironment during infection period (6 h) by host cell inflammatory response even in the absence of additional nitrate or TMAO. Nitrate respiration is suggested to be necessary for *E. coli* growth and colonization in the intestine of mouse; defective nitrate reductase has been shown to greatly reduce colonization of *E. coli* in mouse intestine [23]. Importantly, in this report, the EHEC *narG* single deletion mutant exhibited a deficiency in colonization, as shown by a competitive assay with the wild-type strain. Taking our results into consideration, this could be due to the inefficient maturation of the T3SS machinery resulting in poor establishment of intimate attachment on epithelial cells. Although the inflammatory response is necessary for defense against invading pathogens, inflammation is exploited by some pathogens to outcompete intestinal microflora for efficient infection [14, 24–26].

## Conclusions

The adaptation and usage of the host innate immune response must be an effective strategy for invading pathogens because invading pathogens should compete for limited resources in the intestine with an excess number of microbiota. EHEC may be a successful pathogen due to its acquisition of a nitrate-responding system for the regulation of T3S complex formation. The regulation of the T3SS machinery maturation could be a good target for designing drugs to block EHEC colonization.

## Methods

### Bacterial strains and plasmids

EHEC O157 Sakai (RIMD 0509952) and its derivative SKI1189 (*narGHJI* deletion mutant) [9] were used. Unless otherwise specified, the bacteria were precultured in 1 ml Luria-Bertani (LB) medium overnight at 30 °C, with shaking. Ten milliliter LB medium in a 15-ml tube was inoculated with 100 μl of overnight culture. The bacteria were grown without shaking (standing conditions as microaerobic conditions), in which condition the production of immature T3SS machinery was shown previously [9]. To grow bacteria in the cell culture supernatant, 1 ml of culture in 1.5 ml tubes was incubated in an anaerobic package at 37 °C.

### Analysis of EspB in culture supernatant and whole-cell lysate

Bacteria grown as described above were harvested from 0.5 ml culture by centrifugation and dissolved in SDS-sample buffer (100 μl per OD_600_ unit of original culture). The proteins in the culture supernatant were collected by TCA precipitation (6% TCA) from 8 ml culture supernatant, which was prepared by centrifugation and filtration with a 0.22 mm filter (Millipore). The precipitate was dissolved in SDS-sample buffer (20 μl per OD_600_ unit of original culture). Proteins in 5 μl samples are separated by electrophoresis in SDS-12.5% polyacrylamide gel. Then, the proteins were transferred onto PVDF membrane (Immobilon-P, Merk-Millipore) by using Trans-Blot SD Semi-Dry Transfer Cell (Bio-Rad). EspB protein was detected with anti-EspB antibody [27] and HRP-conjugated anti-rabbit IgG (Cell Signaling Technology). The signals were detected by ECL western blotting detection reagents (Amersham) and ChemDoc MP imager (Bio-Rad).

### Preparation of cell culture supernatant of cytokine-stimulated cells

Confluent Caco-2 cells were prepared 3 weeks before stimulation in a 24-well plate by replacing medium (MEM) every 3–4 days. The cells were incubated with medium containing cytokine mix (2000 U/ml IFN-γ (PeproTech), 50 ng/ml IL-8β and 100 ng/ml IL-22 (Wako pure chemical industries)) for 24 h. When necessary, aminoguanidine hydrochloride (5 mM, TCI Chemicals) was added to the medium. Cell culture supernatant was prepared by centrifugation and filtration.

### Adherence assay

HeLa cell was used for quantification of adherence capacity because of its undifferentiated morphology that was adequate to wash out uninfected bacteria easily and completely. Bacteria were grown overnight in LB at 30 °C, diluted 100-fold in 10 ml of LB medium in 15 ml tube or 1.0 ml of cell culture supernatant in 1.5 ml tube. Bacteria were grown at 37 °C by standing or inside an anaerobic bag for 3–4 h. A semiconfluent monolayer of HeLa cells, which are grown in MEM, on coverslips in a 24-well plate was infected at a multiplicity of infection of 50 for 1.5 h at 37 °C. The cells were washed with PBS and then incubated with fresh DMEM for an additional 3.5 h at 37 °C. After another PBS wash, the cells were fixed and stained with Giemsa. The number of microcolonies, which are clusters containing at least eight bacteria, was determined in three microscopic fields. The number of microcolonies was adjusted for the number of HeLa cells in a given field. Averages and standard errors were calculated from the results of three experiments.

To explore the effect of pre-inflammation, HT-29 cells in 24-well plates were stimulated with heat-killed EHEC (10^8 bacteria/ well) in the presence or absence of aminoguanidine hydrochloride (5 mM) for 2 h before infection. Cells were infected for 3 h, washed 7 times, and then incubated in PBS containing 0.1% Triton X-100 for 20 min at room temperature. After being suspended completely, the viable number of bacteria was measured by spreading part of the diluent (10^-3). Averages and standard errors were calculated from the results of three independent infections.

### Measurement of nitrate concentration

The nitrate concentration was measured by using a NO2/NO3 Assay Kit (Fluorometric) ~ 2,3-Diaminonaphthalene Kit (DOJINDO) following the manufacturer’s instructions.

### Statistical analysis

The statistical significance of differences between groups was examined using an unpaired *t* test. Differences were considered significant at a *P* value of < 0.05.

## Supplementary information

**Additional file 1: Figure S1.** Concentration of nitrate in culture supernatants of Caco-2 cells. **Figure S2.** Original images of Fig. 1b.

## Data Availability

Plasmids will be provided from our lab on request. Raw data can be available by request to TT.

## Abbreviations

T3SS: Type three secretion system
EHEC: Enterohemorrhagic *Escherichia coli*
EPEC: Enteropathogenic *Escherichia coli*
iNOS: Inducible nitric oxide synthase
AG: Aminoguanidine hydrochloride
TMAO: Trimethylamine *N-*oxide
cfu: Colony forming unit
TCA: Trichloroacetic acid
