# Supplementary material for: Enterohaemorrhagic Escherichia coli activates nitrate respiration to benefit from the inflammatory response for initiation of microcolony-formation
Source: BMC Microbiol. 2020 Aug 20;20:261. doi: 10.1186/s12866-020-01946-w (PMC7441704; doi:10.1186/s12866-020-01946-w)
Supplement: Supplementary file 1 — Additional file 1: Figure S1. Concentration of nitrate in culture supernatants of Caco-2 cells. Figure S2. Original images of Fig. 1b. [file 12866_2020_1946_MOESM1_ESM.pdf]

Supplementary information

Enterohaemorrhagic *Escherichia coli* activates nitrate respiration to benefit from the inflammatory response for initiation of microcolony-formation

Risa Nada[1], Shinya Ebihara[1], Hilo Yen, and Toru Tobe*

Department of Biomedical Informatics, Osaka University Graduate School of Medicine,

Osaka, Japan.

1-7 Yamadaoka, Suita, Osaka 565-0871, Japan

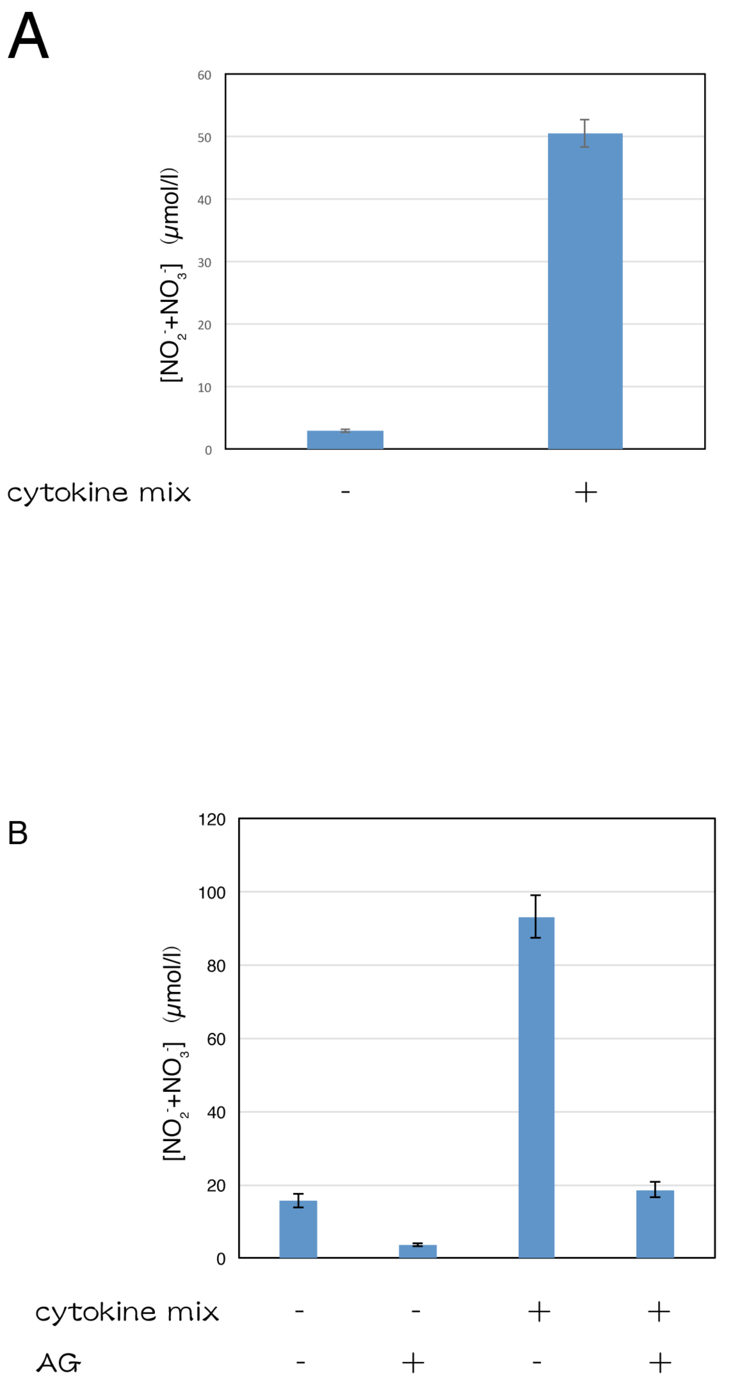

Figure S1. Concentration of nitrate in culture supernatants. A. Nitrate concentration of Caco-2 cells stimulated with cytokine mix. Nitrate concentration was determined after incubation of Caco-2 cells with cytokine mix for 24h. Average and SE (standard error) was calculated from three measurements. B. Effect of aminoguanidine on nitrate concentration. Caco-2 was incubated with cytokine mix along with/without aminoguanidine for 24h. Average and standard error was calculated from three measurements.

EspB in Whole (Fig 1B)

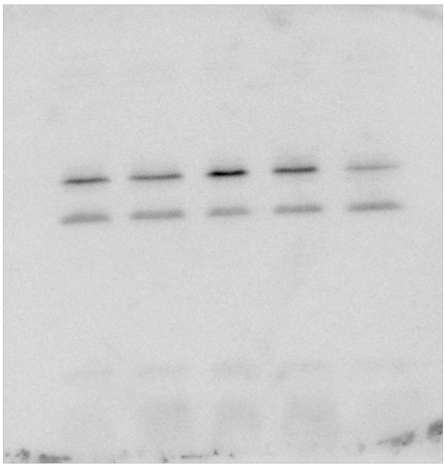

EspB in Sup (Fig 1B)

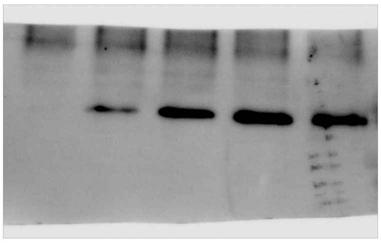

Figure S2. Original images of Figure 1B. Immunoblot with anti-EspB for whole cell proteins (upper panel) and for proteins in supernatant (lower panel).
